# Supplementary material for: Causal Relationship between Aging and Anorexia Nervosa: A White-Matter-Microstructure-Mediated Mendelian Randomization Analysis
Source: Biomedicines. 2024 Aug 16;12(8):1874. doi: 10.3390/biomedicines12081874 (PMC11351342; doi:10.3390/biomedicines12081874)
Supplement: Supplementary file 1 [file biomedicines-12-01874-s001.zip › Supplementary Figure/Figure S4/Singe SNP plot of MR analysis for TL to ubm-b-1813.pdf]

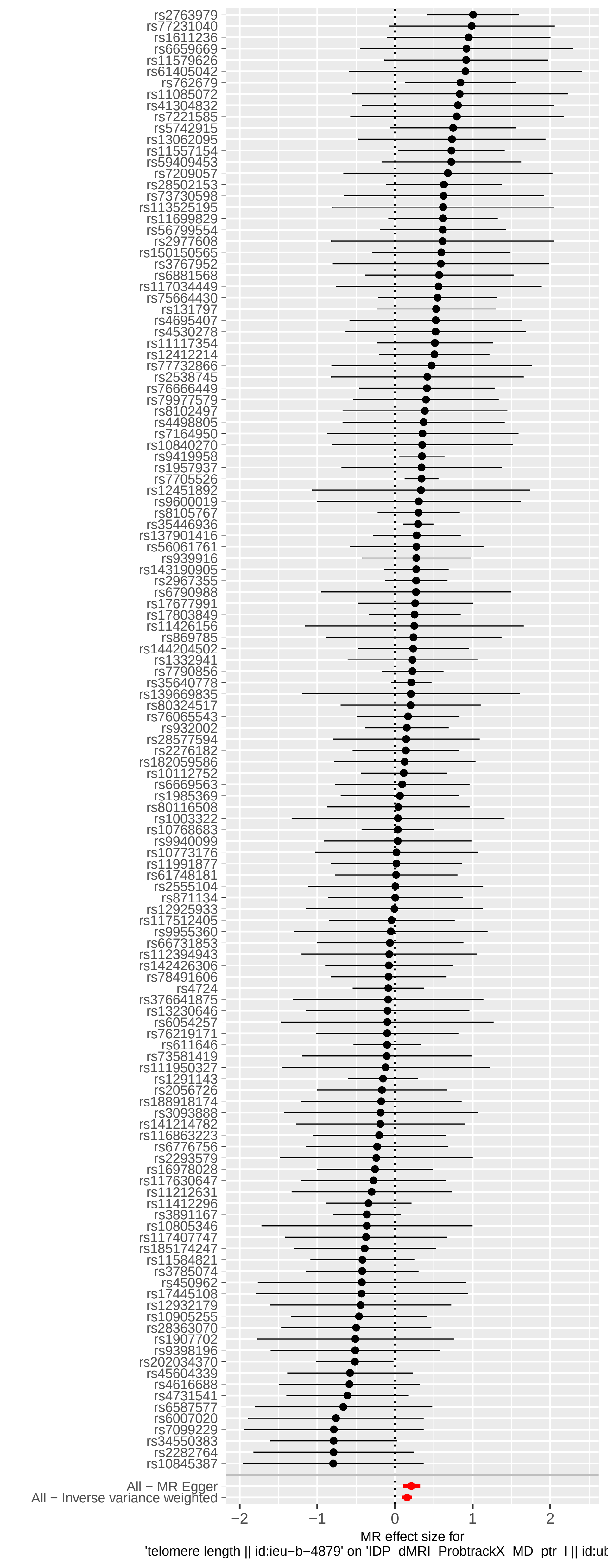
rs2763979
rs77231040
rs1611236
rs6659669
rs11579626
rs61405042
rs762679
rs11085072
rs41304832
rs7221585
rs5742915
rs13062095
rs11557154
rs59409453
rs7209057
rs28502153
rs73730598
rs113525195
rs11699829
rs56799554
rs2977608
rs150150565
rs3767952
rs6881568
rs117034449
rs75664430
rs131797
rs4695407
rs4530278
rs11117354
rs12412214
rs77732866
rs2538745
rs76666449
rs79977579
rs8102497
rs4498805
rs7164950
rs10840270
rs9419958
rs1957937
rs7705526
rs12451892
rs9600019
rs8105767
rs35446936
rs137901416
rs56061761
rs939916
rs143190905
rs2967355
rs6790988
rs17677991
rs17803849
rs11426156
rs869785
rs144204502
rs1332941
rs7790856
rs35640778
rs139669835
rs80324517
rs76065543
rs932002
rs28577594
rs2276182
rs182059586
rs10112752
rs6669563
rs1985369
rs80116508
rs1003322
rs10768683
rs9940099
rs10773176
rs11991877
rs61748181
rs2555104
rs871134
rs12925933
rs117512405
rs9955360
rs66731853
rs112394943
rs142426306
rs78491606
rs4724
rs376641875
rs13230646
rs6054257
rs76219171
rs611646
rs73581419
rs111950327
rs1291143
rs2056726
rs188918174
rs3093888
rs141214782
rs116863223
rs6776756
rs2293579
rs16978028
rs117630647
rs11212631
rs11412296
rs3891167
rs10805346
rs117407747
rs185174247
rs11584821
rs3785074
rs450962
rs17445108
rs12932179
rs10905255
rs28363070
rs1907702
rs9398196
rs202034370
rs45604339
rs4616688
rs4731541
rs6587577
rs6007020
rs7099229
rs34550383
rs2282764
rs10845387
All − MR Egger
All − Inverse variance weighted
−2
−1
0
1
2
MR effect size for
'telomere length || id:ieu−b−4879' on 'IDP_dMRI_ProbtrackX_MD_ptr_l || id:ub
